# Exploring the Anticancer Activity of *Artocarpus heterophyllus* Leaves: Selective Effects on Triple-Negative Breast Cancer and HPV16-Positive Tumorigenic Cells

**DOI:** 10.3390/life15071090

**Published:** 2025-07-11

**Authors:** Ariana Cabrera-Licona, Gustavo A. Hernández-Fuentes, Oscar F. Beas-Guzmán, Alejandra E. Hernández-Rangel, Janet Diaz-Martinez, Osval A. Montesinos-López, José Guzmán-Esquivel, Víctor H. Cervantes-Kardasch, Mario Ramírez-Flores, Alejandrina Rodriguez-Hernandez, Erika R. González-Espinosa, Ana B. Castellanos-Gutiérrez, Francisco Orozco-Ramos, Valery Melnikov, Iván Delgado-Enciso

**Affiliations:** 1 State Cancerology Institute of Colima, Health Services of the Mexican Social Security Institute for Welfare (IMSS-BIENESTAR), Colima 28085, Mexico; arianacabrera267@gmail.com (A.C.-L.); gahfuentes@gmail.com (G.A.H.-F.); francisco.orozco8151@alumnos.udg.mx (F.O.-R.); 2 Department of Molecular Medicine, School of Medicine, University of Colima, Colima 28040, Mexico; oscar.beas.11@gmail.com (O.F.B.-G.); ahernandez157@ucol.mx (A.E.H.-R.); vkardasch@ucol.mx (V.H.C.-K.); mario_ramirez@ucol.mx (M.R.-F.); arodrig@ucol.mx (A.R.-H.); egonzalez65@ucol.mx (E.R.G.-E.); acastellanos21@ucol.mx (A.B.C.-G.); valery.melnikoff@gmail.com (V.M.); 3 Faculty of Chemical Sciences, University of Colima, Coquimatlan 28400, Mexico; 4 Department of Dietetics and Nutrition, Research Center in Minority Institutions, Florida International University (FIU-RCMI), Miami, FL 33199, USA; jdimarti@fiu.edu; 5 Faculty of Telematics, University of Colima, Colima 28040, Mexico; oamontes1@ucol.mx; 6 Clinical Epidemiology Research Unit, Mexican Institute of Social Security, Villa de Alvarez, Colima 28984, Mexico; jose.esquivel@imss.gob.mx; 7 Robert Stempel College of Public Health and Social Work, Florida International University, Miami, FL 33199, USA

**Keywords:** *Artocarpus heterophyllus*, jackfruit, triple-negative breast cancer, cervical cancer, 3D cell culture, phytochemicals, prenylated flavones

## Abstract

*Artocarpus heterophyllus* (jackfruit) is widely distributed in subtropical and tropical regions, and some phytochemicals isolated from this species have demonstrated anti-proliferative effects. However, its impact on triple-negative breast cancer (TNBC) and HPV-related cervical cancer models remains unclear. This study evaluated the phytochemical profile and anticancer activity of an ethanolic extract from *A. heterophyllus* leaves (AHEE) in the TNBC cell line MDA-MB-231 and in the HPV-16^+^ murine cancer cell line TC-1. Phytochemical screening and spectroscopic analyses (UV-Vis, IR, ^1^H, and ^13^C NMR) revealed the presence of tannins, alkaloids, steroids, coumarins, and flavone-type flavonoids, with a total phenolic content of 3.34 µg GAE/mg and flavonoid content of 0.44 mg QE/g extract. In 2D cultures, AHEE reduced cell viability by 49% in TC-1 and 24% in MDA-MB-231 at 300 µg/mL, inhibited colony formation and migration in TC-1, and impaired survival but not migration in MDA-MB-231. In 3D cultures, 250 µg/mL inhibited proliferation, migration, and anchorage-independent growth in both cell lines. Furthermore, the combination of AHEE with one-fifth of the IC_50_ of doxorubicin or cisplatin produces an effect comparable to that observed with the full IC_50_ of these drugs. These findings suggest that AHEE possesses anticancer activity with cell-type-specific effects and highlight its potential as an adjuvant therapy. Further studies are warranted to elucidate its mechanisms of action.

## 1. Introduction

According to the most recent information provided by Global Cancer Observatory (GLOBOCAN) statistics, breast cancer is currently the most common malignancy among women, with 2.3 million cases and 680,000 deaths, while cervical cancer ranks fourth, with 662,000 cases and 349,000 deaths [1].

Breast cancer is the most diagnosed cancer in 183 countries and the leading cause of cancer-related deaths in 169 countries [2,3]. If current trends continue, projections for 2050 estimate a 38% increase in incidence, reaching 3.55 million cases, and a 68% increase in mortality, amounting to 1.14 million deaths [2,4]. This increase can be attributed to delayed diagnosis and the highly heterogeneous nature of the disease, making both diagnosis and treatment challenging for healthcare systems.

In this context, the breast cancer subtype known as triple-negative breast cancer (TNBC) is considered the most aggressive and difficult to treat. TNBC is broadly defined by the absence of estrogen receptors (ERs), progesterone receptors (PRs), and human epidermal growth factor receptor-2 (HER2). Moreover, TNBC is further subdivided into six molecular subtypes, which adds to its complexity [5]. Consequently, it is not responsive to hormonal therapy and has a higher risk of metastasis and recurrence, and a worst prognosis [6]. Surgery remains the primary treatment option, while neoadjuvant therapies include chemotherapy, radiotherapy, and immunotherapy [6]. In chemotherapy, the anthracycline doxorubicin is commonly used, but its effectiveness is limited due to severe side effects and intrinsic or acquired resistance; therefore, new strategies for its administration, as well as its use in combination with other therapies, are under investigation [7].

Concerning cervical cancer, this is the most frequently diagnosed cancer in 25 countries, particularly in Sub-Saharan Africa, South America, and South-Eastern Asia, and remains the leading cause of cancer-related death in 37 countries of these regions [1,3]. Unfortunately, projections for 2050 are not optimistic and estimate that the number of cases will continue to rise to 948,000, and mortality to 543,000 deaths [4].

Approximately 95% of cervical cancer cases are caused by persistent infection with high-risk human papillomavirus (HPV) types [8]. Among these, HPV-16 and HPV-18, of the alpha genus, account for 70% of the cases, although other co-factors also contribute to disease progression [8,9]. In particular, HPV-16 is found in 59.3% of squamous cell carcinomas and 36.3% of adenocarcinomas [8,10].

HPV is primarily transmitted through sexual activity; however, not only the cervix but also other mucosal tissues—such as the vagina, vulva, anus, oropharynx, and penis—are exposed to infection during contact. Therefore, HPV is also associated with extra-cervical malignancies, including oral cancer, making the public health challenge even more complex [10,11]. Although vaccination and regular screening programs have proven effective, their limited implementation in low-income countries contributes to the high incidence of cervical cancer in these regions [12,13]. Surgery is the primary approach for malignant lesions, but in advanced stages, it is typically combined with chemotherapy and radiotherapy [14]. Cisplatin and its derivatives are the first-line chemotherapeutic agents, but their use is constrained by significant side effects and the development of resistance [9,15,16].

A critical issue in oncological research is the ongoing search for new therapeutic options that are not only effective and safe but also minimize adverse effects and act through mechanisms that reduce the risk of drug resistance. Plants have long served as a valuable source of promising anticancer drugs candidates, especially those traditionally used by ancestral populations for medicinal purposes [17]. In this context, characterizing the bioactive chemical constituents of these botanicals is essential to understand and validate their potential therapeutic roles.

One such plant is the jackfruit tree (*Artocarpus heterophyllus*), widely used for its medicinal properties in subtropical regions [18]. Native to India, this evergreen fruit tree is now cultivated across tropical and subtropical regions of America, Asia, and Africa [18]. Traditional medicine utilizes various parts of the plant, including seeds, fruit pulp, bark, wood, roots, and leaves. Jackfruit is rich in bioactive compounds such as lectins, saponins, flavonoids, tannins, carotenoids, and volatile acids, many of which have demonstrated diverse biological activities [18]. For instance, the lectin jacalin, isolated from seeds, has shown anticancer activity in leukemia, breast, and colon cancer cells [19]. Artocarpin (C_26_H_28_O_6_), a prenylated flavonoid derived from wood, has been shown to suppress tumor multiplicity in colon cancer models via downregulation of cytochrome P450 Cyp2c9, and reduce viability in T47D breast cancer cells by activating caspases 3 and 8 [20,21]. Likewise, chromones and flavonoids isolated from the seeds and leaves have demonstrated superior anti-proliferative activity compared to cisplatin in various cancer cell lines, including breast cancer (MCF-7), hepatocarcinoma (SMMC-7721), leukemia (HL-60), pancreatic carcinoma (SW480), and lung cancer (A549) [22]. These findings strongly suggest that *A. heterophyllus* leaves harbor phytochemicals with significant anticancer potential. However, to the best of our knowledge, the effects of their ethanolic extract on triple-negative breast cancer or cervical cancer cells have not yet been explored.

Given the high incidence and mortality of breast and cervical cancer, and the fact that this situation is not expected to improve in the coming decades, the burden on the public health systems in low-income countries will continue to increase. This is further compounded by the challenges in accessing chemotherapeutic treatments or novel therapies, which are often unavailable or unaffordable, as well as the limited information on the potential effects of jackfruit leaf extracts on triple-negative breast cancer (TNBC) and cervical cancer.

To address this, the present study evaluates the anticancer potential of AHEE by analyzing its effects on key biological traits involved in tumor development in TNBC and cervical cancer models. These include characteristics commonly referred to as cancer hallmarks (sustained proliferative signaling, evasion of growth suppressors, resistance to cell death, replicative immortality, angiogenesis, invasion and metastasis, reprogramming of energy metabolism, and immune evasion) [23]. In this work, the MDA-MB-231 cell line was used as a TNBC model, and the TC-1 cell line, a tumorigenic murine model related to HPV-16, was employed as a cervical cancer model. The effects of AHEE on cell viability, proliferation, migration, and anchorage-independent growth in both 2D and 3D culture systems were analyzed to determine its potential anticancer activity. Furthermore, we assessed its potential as an adjuvant therapy by combining it with low doses of doxorubicin and cisplatin and evaluating the combined effect on cell viability.

## 2. Materials and Methods

### 2.1. Preparation of AHEE

Leaves of *Artocarpus heterophyllus* were collected in May 2023, prior to the start of the rainy season, in a remote area of the municipality of Tecoman, Colima, Mexico (18°54′38″ N, 103°52′26″ W/18.910604° N, 103.873789° W). This area features a warm sub-humid climate with seasonal rainfall and is characterized by Cambisol-type soils, which are commonly found in agricultural zones of the region. The collection site was distant from urban or industrial areas, minimizing the risk of environmental contamination.

Species identification was confirmed by comparing the collected specimens with herbarium records housed at the National Herbarium of Mexico (MEXU: 1352538, 179866, and 179865) [24], confirming the material as *A. heterophyllus*. As this species is not listed as protected under Mexican environmental legislation, no special collection permits were required. The harvested leaves were cleaned, air-dried at 38 °C, and pulverized using an industrial-grade blender (Model LM-12, 12 L; Insumos & Equipos JRMR, Cartagena, Colombia) in 60 s cycles. A total of 500 g of powdered leaves were extracted by cold maceration in 2 L of 96% ethanol (AZ, Guadalajara, Mexico) for 24 h under continuous agitation at room temperature using a rotary shaker (HZ-300, JIN YI^®^, Changzhou, China). The resulting mixture was filtered through 2.8 µm Whatman^®^ filter paper (Cytiva, Marlborough, MA, USA), and the solvent was evaporated under reduced pressure at 37 °C for 6 h using a rotary evaporator HB10 (IKA^®^-Werke, Staufen, Germany). The resulting concentrated extract was stored for further analysis.

### 2.2. Phytochemical Characterization and Antioxidant Activity Assessment of AHEE

A preliminary screening was conducted to identify the major classes of secondary metabolites in the ethanolic extract, following Oloya et al. (2021) [25]. Tannins were detected using saturated ferric chloride, while flavonoids were confirmed through reactions with concentrated HCl and magnesium, and the Marini Bettolo test (SbCl_5_ in CCl_4_). Alkaloids were evaluated using Dragendorff’s, Mayer’s, and Wagner’s reagents. Saponins were identified via hemolysis on 7% blood agar and foam formation tests. Steroidal compounds were confirmed by their reaction with H_2_SO_4_ in chloroform. All tests used a 0.1 mg/mL methanolic stock solution of the AHEE.

Total flavonoids were quantified using a colorimetric method adapted from Hernandez-Fuentes et al. (2024) [26]. Extract dilutions from a 0.1 mg/mL methanol stock were reacted with 10% AlCl_3_, 1M potassium acetate, and distilled water. After 30 min of incubation, absorbance was measured at 405 nm. A calibration curve using quercetin (6.25–50 µg/mL) was used to express the flavonoid content as quercetin equivalents (µg/mg of extract).

Antioxidant activity was assessed using the phosphomolybdenum method. The extract was mixed with a reagent containing H_2_SO_4_, NaH_2_PO_4_, and (NH_4_)_6_Mo_7_O_24_, then incubated at 95 °C for 90 min. Absorbance was read at 630 nm, and ascorbic acid was used as a standard. Antioxidant capacity was expressed as a percentage inhibition relative to the control [27].

Reductive power was measured using a modified potassium ferrocyanide–ferric chloride method. The extract was combined with phosphate buffer, K_4_[Fe(CN)_6_], and incubated at 50 °C. After adding trichloroacetic acid and centrifugation, FeCl_3_ was added and absorbance was read at 630 nm. Results were expressed as a percentage reducing power [28].

The Folin–Ciocalteu assay, with adaptations from Hudz et al. (2019) [29] was used to determine total phenolic content (TPC). The extract (0.5 mg/mL) was mixed with diluted Folin reagent and sodium carbonate. After 30 min, absorbance was measured at 765 nm. TPC was calculated using a gallic acid calibration curve (0.005 mg–2 mg/mL) and expressed in mg GAE/100 g of extract [29].

Finally, the antioxidant capacity was also evaluated by DPPH radical scavenging. Different concentrations of the extract (0.005–2 mg/mL) were mixed with a 0.004% DPPH-ethanol solution and incubated in the dark for 30 min. Absorbance was measured at 517 nm. Ascorbic acid served as a positive control. Scavenging activity was calculated using the following formula: Scavenging activity (%) = [A_0_ − (A_x_ − A_γ_)]/A_0_ × 100, where A_0_ = blank, A_x_ = sample, and A_γ_ = control [30].

### 2.3. Thin-Layer Chromatography (TLC) and Spectroscopic Profile

TLC was carried out based on the protocol described by Gwatidzo et al. [31], with slight modifications. Five TLC plates (5 × 5 cm; Silica gel 60 F254, Supelco, Bellefonte, PA, USA) were prepared by drawing a baseline 0.5 cm from the bottom edge using a pencil. Extract samples were applied to this line using micropipettes, depositing 1 µL of each sample at a concentration of 50 mg/mL. For comparison, three standard compounds—quercetin (Essential Nutrition, Monterrey, Mexico), 4-methylumbelliferone, and anthrone (Sigma-Aldrich, St. Louis, MO, USA)—were also applied at 1 mg/mL.

Chromatographic separation was performed using either pure chloroform or a chloroform/methanol (CCl_3_/MeOH) mixture as the mobile phase in a development chamber. After development, the solvent front was immediately marked. The plates were examined under ultraviolet light at wavelengths of 254 and 365 nm. Subsequently, different visualization reagents were applied, including ceric sulfate, 1% ferric chloride, and 1% ethanolic aluminum chloride spray. The resulting chromatograms were photographed, and retention factor (Rf) values were calculated as the ratio of the distance traveled by the compound to the distance traveled by the solvent front (Rf = compound distance/solvent front distance), as shown in Figure S1. Each analysis was performed in triplicate to ensure reproducibility [32].

UV spectra of the AHEE stock solution (0.1 mg/mL in methanol) were recorded using an Evolution 300 spectrophotometer, with methanol as the solvent. FTIR data were collected using a Shimadzu FTIR-TRACER-100 spectrophotometer. A preliminary NMR analysis, including ^1^H, ^13^C, DEPT-90, and DEPT-135 experiments, was performed on a Bruker 400 MHz spectrometer (Leipzig, Germany) using dimethyl sulfoxide-d6 (DMSO-d6, Sigma-Aldrich, Saint Louis, MO, USA) as the solvent. Chemical shifts (δ, ppm) and coupling constants (J, Hz) were recorded. The spectral data obtained from UV, IR and NMR are shown in Supplementary Files (Figures S2–S6). The NMR shifts obtained were compared with published NMR data of isolated compounds from *A. heterophyllus* leaves (see Supplementary Table S1) [33,34,35,36].

### 2.4. Evaluation of Anti-Browning Effects on Fresh-Cut Apple Slices

To assess the anti-browning potential of the ethanolic extract of AHEE, a modified version of the protocol described by Lee et al. (2023) was employed [37]. Apple slices (*Malus domestica* ‘Starking Delicious’) of consistent size were immersed in one of the following treatments: a solvent control (ethanol/water), a 0.5 mg/mL ascorbic acid solution (used as positive control) [38], or a 0.5 to 1.0 mg/mL AHEE solution. All treated slices were kept at 20 °C, and browning was documented at 0, 12, 24, 36, and 48 h. Changes in color parameters (L*, a*, and b*) were analyzed using CorelDRAW software (version 25.0, US), based on the CIE color system. The total color variation (∆E) was calculated using the following formula: (∆E)^2^ = (L − L_0_)^2^ + (a − a_0_)^2^ + (b − b_0_)^2^ [37].

### 2.5. Preparation of Cell Treatments

The AHEE was prepared at 10 mg/mL by dissolving in sterile water at 37 °C with 1000 rpm shaking (ThermoMixer^®^, Eppendorf, Hamburg, Germany) and filtering with a 0.22 µm membrane (TPP^®^, Trasadingen, Switzerland). For the treatments, a 1 mg/mL dilution was prepared in Dulbecco’s Modified Eagle’s Medium-High Glucose, DMEM-HG, (Biowest^®^, Bradenton, FL, USA), supplemented with 1X penicillin/streptomycin/amphotericin B (Antibiotic-Antimycotic 100X, Biowest^®^, Bradenton, FL, USA). Low concentrations of 1, 15, 25, 50, 75, and 100 µg/mL, and higher concentrations of 150, 200, 250, and 300 µg/mL, were tested in DMEM-HG supplemented with 1% (*v*/*v*) calf serum (Biowest^®^, Bradenton, FL, USA). Fresh dilutions of the extract were used for each assay. The concentrations tested correspond to parameters used to define if an extract is highly active (<100 µg/mL) or active (100 to 500 µg/mL) [39].

### 2.6. Cell Culture

The TNBC MDA-MB-231 cells (ATCC: HTB-26, Manassas, VA, USA) and tumorigenic murine cervical cancer cell line TC-1 ([Mouse lung] (RRID: CVCL_4699), https://www.cellosaurus.org/CVCL_4699, accessed on 20 July 2024) were used for all assays. The TC-1 line is a recognized model for in vivo studies of cervical cancer and other HPV-related cancers. It is genetically engineered to express the HPV-16 oncogenes E6 and E7 and the human c-Ha-ras oncogene, making it tumorigenic in mice [40]. It is therefore widely used in the field of vaccine development research against this cancer, as it mimics the local and systemic antitumor immune response [41,42,43,44,45].

The cell lines were grown in a 25 cm^2^ tissue culture flask with a filter screw cap PTFE membrane having a 0.22 μm pore size (TPP^®^, Trasadingen, Switzerland) and maintained in 1X DMEM-HG medium (Biowest^®^, Lakewood Ranch, FL, USA) supplemented with 10% (*v*/*v*) calf serum (Biowest^®^, Lakewood Ranch, FL, USA) and 1X penicillin/streptomycin (Biowest^®^, Lakewood Ranch, FL, USA) in a humidified atmosphere (95% air and 5% CO_2_) at 37 °C (ESCO CelCulture^®^ Incubator, Hatboro, PA, USA). For subculturing, 0.025%Trypsin-EDTA Solution (GibcoTM, Life Technologies Corp., Grand Island, NY, USA) was used.

### 2.7. Cell Viability Assay

Cells at a density of 1 × 10^4^ cells/100 µL/well were seeded in TC-treated 96-well flat-bottom microplate (TPP^®^, Trasadingen, Switzerland) with maintenance medium and under normal incubation conditions for 24 h. Afterward, the medium was replaced with 100 µL/well of each treatment, and the microplate was incubated for 48 h in standard conditions. As a blank control, DMEM-HG medium supplemented with 1% calf serum was used. As a positive anticancer control, doxorubicin (Teva Pharma AG, Basel, Switzerland) was used at a concentration of IC_50_ for 48 h of 2 µM (1.159 mg/mL) for the assays with MDA-MB-231, and cisplatin (ACOCIT^®^ Intas Pharmaceuticals Limited, Matoda, India) at a concentration of IC_50_ for 48 h of 58.3 µM (17.4 mg/mL) for the assays with TC-1 cells [46,47].

After the treatment period, images were captured with the AE31 EPI inverted microscope coupled to the Moticam Pro 252A camera and with Motic Images Plus 2 software (Motic^®^, Xiamen, China). Later, the medium was replaced with 100 µL/well 1X Alamar Blue™ Cell Viability Assay Reagent (Sigma-Aldrich, Saint Louis, MO, USA) in phenol red-free medium. The microplate was incubated for 4 h in the dark under standard conditions. Colorimetric changes due to the reduction of resazurin (blue) to resorufin (pink), by metabolically active cells, were measured. Absorbance was monitored at 570–600 nm with a microplate reader (iMarkTM, Bio-Rad, Hercules, CA, USA) according to the manufacturer’s protocol. The viability index was calculated as follows: (Ab_570/600_ value experimental—Ab_570_/_600_ value blank)/(Ab_570_/_600_ value control—Ab_570/600_ value blank) × 100% [48].

### 2.8. Clonogenic Assay

Cells at a density of 3 × 10^3^ cells/500 µL/well were seeded in a TC-treated 24-well flat-bottom plate (TPP^®^, Trasadingen, Switzerland) in a maintenance medium and under standard incubation conditions for 24 h. Then, the medium was replaced with 500 µL/well of the treatments and controls, and the plate was incubated for 24 h under standard conditions. At the end of the exposure time, the treatments were replaced with fresh maintenance medium, and the plate was incubated for 10 days with medium changes every 4 days [48]. Finally, cells were fixed with 10% buffered formalin, stained with 0.5% crystal violet for 45 min, washed with H_2_O until the excess dye was removed, and allowed to dry. Photographs were taken of each treatment condition. For quantification, the dye was extracted with 10% acetic acid incubation for 1 h with shaking [49]. Differences in dye uptake were measured at a 570 nm wavelength (BioMate^TM^, Thermo, Madison, WI, USA) and the survival percentage was calculated as follows: % = (treatments OD × 100)/control OD, where OD is the optical density at 570 nm.

### 2.9. Wound Healing Assay

Cells were seeded at a density of 5 × 10^5^ cells/500 µL/well in a TC-treated 24-well flat-bottom plate (TPP^®^, Trasadingen, Switzerland) in the maintenance medium and incubated under standard parameters until they reached approx. 90% confluence. Then, monolayers were scraped using a 200 μL tip, washed with 1X PBS sterile at pH 7.4, and exposed to serum-free treatments. Images of the wounded area were captured immediately thereafter, and this was considered time zero. The plate was incubated under standard conditions for 24 h, and then cell migration in the wounded area was captured in images. Analysis of the changes in the edges of the wound area was performed using TScratch 1.0 software (https://github.com/cselab/TScratch, accessed on 7 July 2025) [50]. Area reduction was estimated as follows: % = 100−[(area T_x_ × 100)/area T_0_], where T_x_ = the area at 24 h and T_0_ = the area at baseline.

### 2.10. Anchorage-Independent Growth Assay (Spheroid Formation Inhibition Assay)

The cells at a density of 5 × 10^3^ cells/100 µL/well resuspended in the respective treatments or control medium were seeded in a non-adherent surface created by 1% agarose in a 96-well flat-bottom microplate (TPP^®^, Trasadingen, Switzerland) and incubated under standard conditions for 48 h [51,52]. Then, treatments were replaced with 100 µL of complete medium and microplates were incubated for 48 h to enable cell grouping. Images were captured at this time point. To evaluate the effects on cells, these were transferred to a TC-treated 96-well flat-bottom microplate (TPP^®^, Trasadingen, Switzerland) and incubated for 72 h. Images were captured, and the adhesion/migration/proliferation differences were determined by measuring expansion area with ImageJ 1.54 software (NIH, Bethesda, MD, USA) and calculating fold expansion as (migration area Treatment × 1)/migration area Control [53].

### 2.11. Three-Dimensional Culture Formation and Reversion to Two-Dimensional Culture

Three-dimensional cultures (spheroids) were performed by the liquid overlay technique [54,55]. The cells at a density of 5 × 10^3^ cells/100 µL/well were seeded in a non-adherent surface created by 1% agarose in a 96-well flat-bottom microplate (TPP^®^, Trasadingen, Switzerland) in maintenance medium and incubated under standard parameters for 96 h. Images were captured to establish time zero, the medium was carefully replaced with the treatments and controls, and the plate was incubated under standard conditions for 48 h. At the end of the exposure time, images were captured, and spheroids were transferred to a TC-treated 96-well flat-bottom microplate (TPP^®^, Trasadingen, Switzerland) and incubated for 72 h, to evaluate the reversion to 2D cultures, and images were captured at this time [56]. The spheroid reversion/migration area measurements were performed with ImageJ 1.54 software (NIH, Bethesda, MD, USA). The differences in the reversion/migration were calculated as follows: Fold expansion = (migration area Treatment × 1)/migration area Control [53].

### 2.12. Interaction Assays Between AHEE and Chemotherapeutics

The interaction between AHEE, doxorubicin, and cisplatin was analyzed by determining changes in cell viability. Cell lines were seeded as previously described in the cell viability assay Section 2.7. The MDA-MB-231 cells were treated for 48 h with combinations of 1/5th of the IC_50_ of doxorubicin (0.40 μM = 0.23 mg/mL) and 100, 150, 200, 250, and 300 µg/mL of AHEE. The TC-1 cells were treated for the same time with the combination of the extract concentrations and 1/5th of the IC_50_ of cisplatin (11.66 μM = 3.48 mg/mL). The IC_50_ values of chemotherapeutics and medium alone were included as controls. Cell viability was quantified as previously described [57].

### 2.13. Statistical Analysis

The graphs show the mean ± standard error of the mean (SEM) [58]. In all cases, the results are representative of three independent experiments. Ten replicates per experiment were performed in the case of viability assays and 3D culture assays, and three replicates in the clonogenic and wound healing assays. One-way ANOVA and Tukey’s post hoc multiple comparisons test was applied to analyze differences between treatments at a significance level of *p* < 0.05 [59]. Analyses were performed with the statistical program GraphPad Prism version 8.0.1 for Windows (GraphPad Software, San Diego, CA, USA.

## 3. Results

### 3.1. AHEE Is Rich in Flavones and Exhibits a Moderate Antioxidant Capacity

The extract was obtained from 500 g of leaves, yielding 49 g of a dark green semi-solid material, representing a yield of 12.25%. Table 1 shows that AHEE contains appreciable amounts of tannins, steroids, and alkaloids, with moderate to trace levels of coumarins and chalcone-type flavonoids. The extract yielded a total flavonoid content of 0.45 mg QE/g and a phenolic content of 3.34 µg GAE/mg. Antioxidant assays revealed a DPPH radical scavenging effect of 86.34%, total antioxidant capacity of 81.25%, and ferric reducing power of 34.09%.”

### 3.2. Chromatographic and Spectroscopic Analysis of AHEE

Thin-layer chromatography (TLC) was performed using the best conditions of separations for the standards employed. The mobile phase consists of chloroform:methanol (8:2, *v*/*v*) to profile the phenolic constituents of AHEE. Three reference standards were included for comparison: quercetin (Q, Rf = 0.59), anthrone (A, Rf = 0.95), and 4-methylumbelliferone (Rf = 0.77). In the AHEE lane, several spots were observed, among which a prominent band at Rf = 0.74 was particularly noteworthy. This band exhibited strong fluorescence under long-wave UV light (365 nm), emitting a bright pink coloration reminiscent of Bengal rose. This intense pink fluorescence may be indicative of coumarin or flavonoid derivatives with extended conjugation, which are known to display such optical behavior under UV light due to their π-π* electronic transitions [60,61].

UV-Vis spectroscopy of the extract revealed absorption maxima around 224, 278, 327, and 412 nm, consistent with aromatic and phenolic compounds. Infrared (IR) spectroscopy showed characteristic bands at 1043, 1438, 1517, 1604, 1734, 2850, 2919, 2956, and 3236 cm^−1^, indicating the presence of hydroxyl, aromatic, carbonyl, and aliphatic functional groups [33,62,63].

The nuclear magnetic resonance (NMR) spectra of AHEE confirmed a complex chemical profile. The ^1^H NMR spectrum displayed multiple singlets in the aromatic region (δH 11.01–6.21 ppm), characteristic of hydroxylated aromatic rings typical of polyphenolic compounds such as flavonoids. The aliphatic region showed various multiplets and singlets (δH 5.30–0.78 ppm), indicative of methylene, methyl, and possible methoxy groups. The ^13^C NMR and DEPT experiments revealed prominent signals in the downfield region (δC > 160 ppm), consistent with carbonyl and conjugated aromatic carbons, as well as upfield signals (δC < 40 ppm) attributed to aliphatic carbons. Additionally, signals within δC 50–60 ppm may correspond to methoxy substituents [64,65].

Table S1 presents a comparative analysis of the chemical shifts of AHEE alongside those of several secondary metabolites previously isolated from *A. heterophyllus* leaves, including asterric acid, licoflavone C, artocarpin, artocapone, and artocarpetin. Notably, the chemical shifts of AHEE show strong similarity to artocarpin and artocarpetin, particularly in the aromatic and carbonyl regions (e.g., δC ~180.3, 163.1, 159.9, and 122.0 ppm), as well as in the aliphatic regions related to methyl and methoxy groups [33,34,35,36].

Considering the data in Table S1, a marked resemblance is observed between some of the signals in AHEE and those of the flavonoids mentioned. Although additional experiments such as compound isolation and 2D NMR are required for definitive identification, these spectral similarities suggest the possible presence of such bioactive compounds within the extract. This preliminary comparison supports the hypothesis that AHEE contains flavonoid-type metabolites similar to those previously reported in *A. heterophyllus*, underscoring the phytochemical potential of this plant and warranting further detailed studies.

### 3.3. AHEE Antioxidant Potential

**Figure 1:**
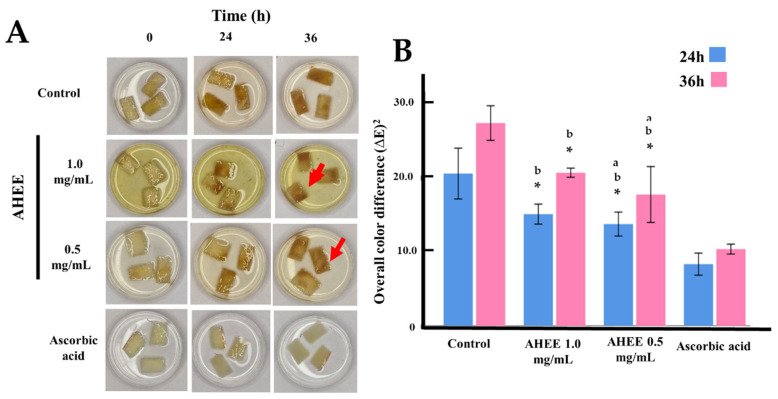
Anti-browning activity of AHEE. (**A**) Representative images showing the effect of AHEE on browning of freshly cut apple slices over 24 and 36 h at 20 °C. Ascorbic acid (5 mg/mL) was used as the positive control of antioxidant activity. All samples were treated in an aqueous solution. Each storage time point shows photographs of apple slices treated with AHEE at 0.5 mg/mL and 1.0 mg/mL. The red arrows indicate softening. (**B**) Graphical representation of the overall color difference (∆E)^2^, calculated as follows: (∆E)^2^ = (a—a initial)^2^ + (b—b initial)^2^ + (L—L initial)^2^. * Statistical difference compared to ascorbic acid (*p* < 0.05); ^a^ Statistical difference compared to AEEE 1.0 mg/mL (*p* < 0.05). ^b^ Statistical difference compared to control (*p* < 0.05).

Table 2 and Figure 1 present the anti-browning effects of AHEE on freshly cut apple slices over 36 h at 20 °C, measured as the overall color difference (∆E). The mean ∆E values ± standard deviation indicates that both concentrations of AHEE (1.0 and 0.5 mg/mL) significantly reduced enzymatic browning compared to the untreated control at both 24 and 36 h (*p* < 0.05). Ascorbic acid (5 mg/mL), used as a positive control, exhibited the strongest anti-browning effect with the lowest ∆E values (8.28 ± 1.48 at 24 h and 10.28 ± 0.67 at 36 h). At 24 h, AHEE at 1.0 mg/mL and 0.5 mg/mL showed similar efficacy (∆E = 14.98 ± 1.60 and 13.64 ± 1.60, respectively; *p* = 0.812). At 36 h, ∆E values increased for all treatments, yet AHEE concentrations remained significantly more effective than the control (*p* < 0.05), although less potent than ascorbic acid.

### 3.4. AHEE Induces Changes in the Morphology of MDA-MB-231 and TC-1 Cells, Decreases Their Viability, and Inhibits Their Ability to Proliferate

**Figure 2:**
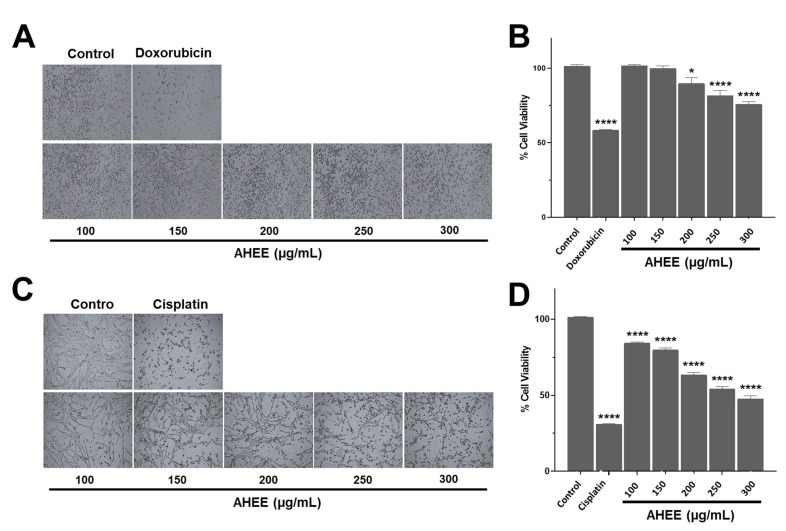
Morphological and cell viability changes in TNBC and tumorigenic HPV-16+-related cell lines induced by AHEE. (**A**) The microphotographs show the normal morphology of MDA-MB-231 cells of TNBC and the morphological changes in those treated with IC_50_ doxorubicin. In the cultures treated with the extract for 48 h, rounded cell morphology appeared after 48 h exposure starting at 200 µg/mL. Magnification 4×. (**B**) The viability of this cell line was slightly affected, with a decrease of 24% at the highest extract concentration of 300 µg/mL. (**C**) The microphotographs show the normal morphology of tumorigenic HPV-16+-related TC-1 cells and the morphological changes in those treated with IC_50_ cisplatin. Shortening of the cell prolongations was observed from a concentration of 150 µg/mL and increased in a concentration-dependent manner. Magnification 4×. (**D**) The viability of this line declined in a concentration-dependent manner up to 51% at the highest tested concentration of AHEE. * *p* < 0.05, **** *p* < 0.0001, compared to the control with medium alone.

Initial screening was performed with concentrations of 1, 15, 25, 50, 75, and 100 µg/mL. At these low concentrations, a slight decrease in viability was observed at 100 µg/mL, so it was decided to try higher concentrations. Screening at concentrations of 100, 150, 200, 250, and 300 µg/mL revealed a concentration-dependent effect (Figure 2).

Morphological changes were observed in both cell lines. These cells present an elongated fibroblastoid-like morphology and form a network-like monolayer. When they were exposed to the extract for 48 h, a concentration-dependent shortening of the cell prolongations was evident. In MDA-MB-231 cells, these changes began at 200 µg/mL and were mild (Figure 2A), whereas in the TC-1 line, the effect was visible from 150 μg/mL and was more markedly increasing than in TNBC cells, and almost similar to the damage caused by the IC_50_ dose of cisplatin (Figure 2C).

These changes in morphology corresponded to a gradual decrease in cell viability, being moderate in MDA-MB-231 and more pronounced in TC-1 cells. The AHEE at 100–150 µg/mL did not affect MDA-MB-231 cells, but at 200 µg/mL, viability decreased by 10.14% ± 3.79, and this was statistically different from the untreated control (*p* = 0.0151), at 250 µg/mL by 18.52% ± 3.54 (*p* < 0.0001) and at 300 µg/mL by 24.09% ± 1.70 (*p* < 0.0001); however, they did not reach the level of reduction of IC_50_ of doxorubicin, which was 41.53% ± 0.38 (Figure 2B).

In contrast, from 100 µg/mL in TC-1 cells the effect was statistically different to the control with a reduction of cell viability of 15.79% ± 0.72; at 150 µg/mL, of 20.38% ± 1.39; at 200 µg/mL, of 36.64% ± 1.61; at 250 µg/mL, of 45.92% ± 1.67; and at 300 µg/mL, of 52.45% ± 2.14 (*p* < 0.0001, in all the cases). The latter concentration was close to the effect of cisplatin on viability, which was a 68.98% ± 0.36 reduction (Figure 2D). In other words, the maximum concentration of the extract tested in the TNBC line only induced a reduction equivalent to almost half of doxorubicin IC_50_, while in the HPV-16-related cell line, its effect on viability was comparable to the effect caused by cisplatin.

**Figure 3:**
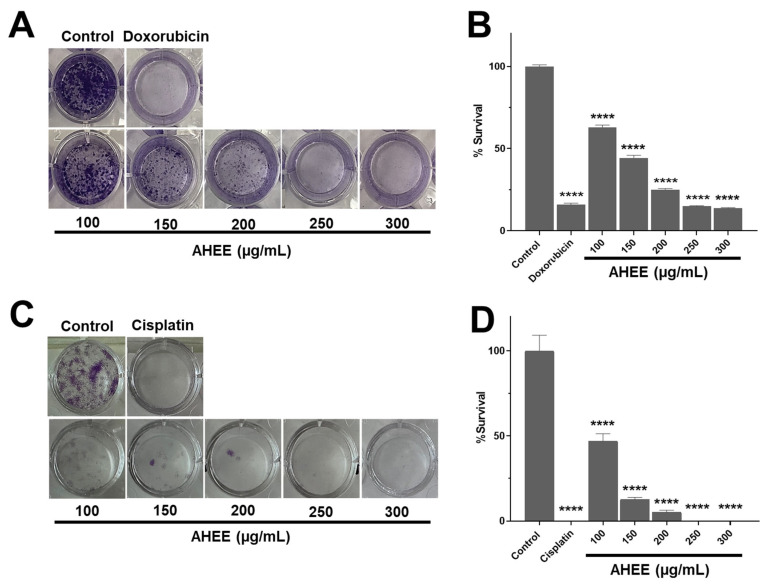
Changes in proliferation/survival in TNBC and tumorigenic HPV-16+-related cell lines induced by AHEE determined by clonogenic assay. (**A**) The ability of MDA-MB-231 cells to form colonies, divide, and proliferate decreased in a concentration-dependent manner when treated with the extract for 24 h compared to the control and had a similar effect to doxorubicin. (**B**) The percentage of survival gradually diminished up to 85% at the highest extract concentration tested of the 300 µg/mL extract. (**C**) In TC-1 cells, the impact on division and proliferation was more marked, until no colonies were observed at the concentration of 200 µg/mL of the extract. (**D**) At 200 µg/mL, survival declined up to 95% and was fully inhibited at 250–300 µg/mL, similarly to cisplatin. **** Statistical difference compared to the control with medium alone (*p* < 0.0001).

These data show that the AHEE exhibits negative regulatory activity against the cancer hallmark of cell death evasion/immortality. Although a concentration of 300 µg/mL nearly induced 50% cell death in the TC-1 cells, higher concentrations were not tested to achieve 100% cell death, as our goal was to determine whether AHEE was highly active (<100 µg/mL) or active (100 to 500 µg/mL) [39]. When we analyzed the effect of the extract on cell proliferation by clonogenic assays (the ability to form colonies), a similar cancer-type-specific activity was observed, that is, it was more marked in TC-1 cells than in MDA-MB-231 cells (Figure 3). At 100 µg/mL, MDA-MB-231 survival was 62.99%, that is a reduction of 37.01% ± 1.33; at 150 µg/mL, the reduction was 55.62% ± 1.53; at 200 µg/mL, it was 74.89% ± 0.63; at 250 µg/mL, it was 85.11% ± 0.39; and at 300 µg/mL, it was 86.30% ± 0.41; all of these were statistically different from the control with *p* < 0.0001 (Figure 3A,B). The effects at 250 and 300 µg/mL were statistically comparable to those of doxorubicin, which decreased survival by 84.08% ± 1.90, so they had a similar effect (*p* = 0.9873 and o = 0.6763, respectively).

In contrast, the percentage of cell survival decreased drastically in TC-1 cells at 100 µg/mL to 53.16% ± 4.56, at 150 µg/mL to 87.34% ± 1.27, and at 200 µg/mL to 94.94% ± 1.27, and was fully inhibited at 250 and 300 µg/mL, showing no statistical difference compared to cisplatin, which also inhibited proliferation (*p* > 0.9999) (Figure 3C,D).

It should be noted that these tests were carried out with only 24 h of exposure to the extract to rule out the decrease in the number of colonies from being attributable to cell death. This result shows that AHEE leaves interfered more specifically with the cancer hallmark of the ability to proliferate indefinitely, so this may have a specific modulatory activity on signaling pathways or cellular mechanisms that allow the proliferation/survival of malignant cells.

### 3.5. AHEE Impacts the Migration of MDA-MB-231 and TC-1 Cells

**Figure 4:**
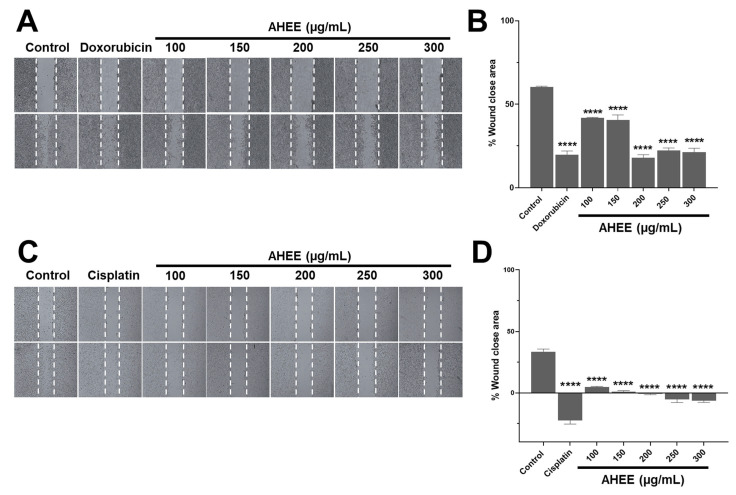
Changes in migration in TNBC and tumorigenic HPV-16+-related cell lines induced by AHEE. (**A**) The elongation of MDA-MB-231 cells at the wound edges was evident in untreated monolayers, as well as in the 100 and 150 µg/mL concentrations of the extract, while prolongation shortening was evident in those treated with doxorubicin and at concentrations of 200–300 µg/mL of the extract. Magnification 4×. (**B**) Quantification of the percentage of wound area closure showed that doxorubicin and higher concentrations of the extract have similar action, with a reduction of only approximately 20%. (**C**) In the untreated TC-1 monolayers, the prolongation of the cells from the wound edges was observed. In contrast, in all monolayers treated with the extract, the shortening of the prolongations was obvious, as with cisplatin, which also induces rounding of the cells, and therefore a larger wound area. Magnification 4×. (**D**) The quantification of the wound closure area showed negative values equivalent to an increase in the area up to 6% at 300 µg/mL of AHEE and up to 22% in cisplatin. **** *p* < 0.0001, compared to the control with medium alone.

Another distinctive hallmark of cancer is the ability of cells to migrate and invade other tissues to generate metastasis. In this regard, wound healing assays revealed that AHEE strongly inhibited of TC-1 cells but had a limited effect on MDA-MB-231 cells (Figure 4). This assay was performed by exposing the monolayers for 24 h to treatments in free-serum media to minimize the cell proliferation.

Specifically, in the control MDA-MB-231 monolayer, the wound edges closed 60.31% ± 0.48 of the original wound area. In contrast, cells treated with AHEE at 100 µg/mL showed 41.85% ± 0.29 closure; at 150 µg/mL, 40.45% ± 3.10 closure; at 200 µg/mL, 18.00% ± 1.73 closure; at 250 µg/mL, 22.32% ± 1.50 closure; and at 300 µg/mL, 21.23% ± 2.33 closure; all were statistically different from the control (*p* < 0.0001).

Compared to doxorubicin-treated monolayers, which exhibited 19.73% ± 4.45 closure, the wound closure values for 200, 250, and 300 µg/mL AHEE were not significantly different, with *p*-values of 0.9943, 0.9575, and 0.9975, respectively (Figure 4A,B). Overall, the highest concentrations of AHEE reduced migration by nearly two-thirds, an effect comparable to doxorubicin.

The AHEE-induced changes in TC-1 cell monolayers were more pronounced. At 100 µg/mL, the wound area was reduced by only 4.69% ± 0.62, and at 150 µg/mL by 0.93% ± 0.93. Notably, at concentrations of 200, 250, and300 µg/mL, cells became rounded, causing wound edge contraction, which was reflected as an increase in the wound area: 1% ± 0.49 at 200 µg/mL; 5.35% ± 2.54 at 250 µg/mL; and 6.33% ± 1.50 at 300 µg/mL. These changes were statistically significant compared to the control group, which showed a wound area reduction of 33.34% ± 2.32 (*p* < 0.0001, in all the cases), and also compared to cisplatin-treated cells, in which the wound area increased to 22.44% ± 5.26 (Figure 4C,D).

We confirmed that these effects in TC-1 cells were independent of cell death by staining monolayers with the supravital dye trypan blue. No positive (dead) cells were detected in either the treated or control conditions, with very few positive cells observed only in the cisplatin-treated monolayers. Taken together, these results suggest that AHEE directly affects cell migration.

### 3.6. Exposure of MDA-MB-231 and TC-1 Cells to AHEE Impacts Their Ability of Anchorage-Independent Growth

Another important characteristic of cancer cells is their ability to grow anchorage independently to migrate and invade other tissues [52,66]. To analyze this point, we treated cells with AHEE and seeded them on a non-adherent surface to determine whether they could aggregate and form 3D cultures (spheroids).

**Figure 5:**
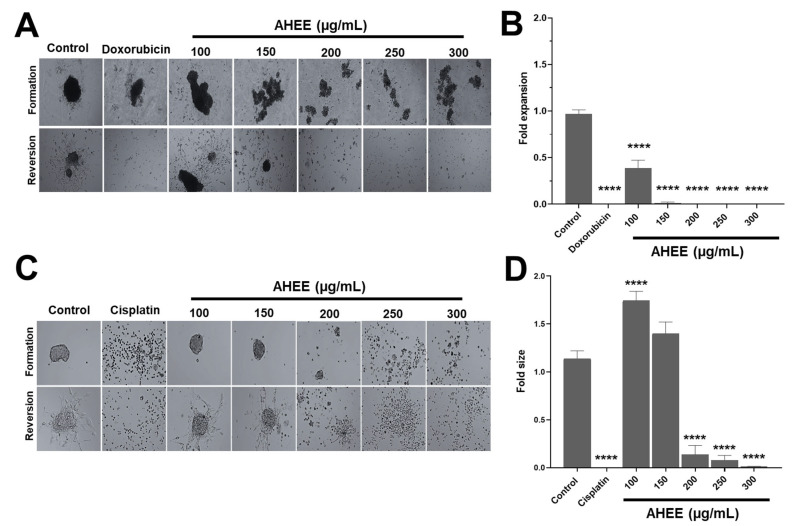
Changes in anchorage-independent growth of TNBC and tumorigenic HPV-16+-related cell lines induced by AHEE. (**A**) Untreated MDA-MB-231 cells clustered into spheroids on a non-adherent surface, as well as those treated with 100 µg/mL of the extract for 48 h, and those exposed to doxorubicin. In contrast, from 150 µg/mL of extract, they formed small clusters. Magnification 4×. (**B**) Cells were transferred to an adherent surface, and the area of expansion to 2D culture was measured. At 200 µg/mL of the extract, the ability of the cells to adhere, proliferate, and migrate was inhibited similarly to doxorubicin treatment. (**C**) TC-1 cells formed well-defined spheroids, similar to the control, at 100 and 150 µg/mL of treatment, but at 200 µg/mL and above the cell clusters became increasingly smaller and more dispersed. Magnification 4×. (**D**) Quantification of the area of reversion to 2D cultures showed that at 200 µg/mL and above, the ability of cells to adhere, proliferate, and migrate decreased abruptly, similar to cisplatin treatment. **** *p* < 0.0001 compared to the control with medium alone.

Untreated MDA-MB-231 cells successfully aggregated into spheroids, as did those exposed to 100 µg/mL of AHEE for 48 h. In contrast, from 150 µg/mL of AHEE, smaller cell aggregates were observed that did not organize into a spheroid form (Figure 5A). Subsequently, it was evaluated whether these cells maintained the ability to adhere, proliferate, and expand/migrate on an adherent surface.

To quantify the impact on these characteristics, the expansion area of cells on the adherent surface was measured and compared with that of control cells. Under these criteria, 100 µg/mL of AHEE affected the ability of the cells to grow in an anchorage-dependent manner, because it had an average expansion of 0.39 ± 0.08, corresponding to approximately one-third of the control cell area. The treatment of 150 µg/mL of AHEE decreased the expansion value to 0.015 ± 0.01, and the subsequent concentrations of 200–300 µg/mL completely inhibited cell expansion. Statistical analysis showed that, versus control, all AHEE treatments were significantly different with a *p*-value of <0.0001. In comparison with doxorubicin, 150 µg/mL of AHEE had a *p*-value of 0.9982, and concentrations of 200–300 µg/mL had *p*-values > 0.9999, that is, they had a similar effect (Figure 5B).

In contrast, when TC-1 cells were treated with 100–150 µg/mL of AHEE, they formed cell aggregates in the same way as the untreated control cells, while on the adherent surface, they presented an expansion area also similar to the control (Figure 5C). However, at 200 µg/mL and above, the cell clusters were small and dispersed. On an adherent surface, the aggregated cells treated with 200 µg/mL had an expansion area of 0.14 ± 0.09, the cells treated with 250 µg/mL of AHEE had an area of 0.08, and cells treated with 300 µg/mL had an expansion area of 0.01. These values were statistically different from the control (*p* < 0.0001 in all cases); however, in comparison with cisplatin, they did not differ, with a *p*-value of 0.7979 vs. 200 µg/mL of AHEE, *p* = 0.9849 vs. 250 µg/mL, and a *p*-value of >0.9999 vs. 300 µg/mL (Figure 5D).

These results suggest that AHEE interferes with the anchorage-independent growth capacity, as the aggregates formed became smaller and smaller. Furthermore, we subsequently found that the cells in the aggregates were impaired in their ability to grow in culture and in their viability, corroborating the effect of AHEE.

### 3.7. Exposure of TC-1 and MDA-MB-231 Cells’ 3D Cultures to AHEE Impacts Their Ability to Proliferate and Migrate

Three-dimensional spheroids, or 3D cell cultures, are a study model that has emerged in recent years and is gradually becoming more widely used due to its advantages, as they are used to screen anticancer drugs, since they mimic some tumor conditions in comparison to an in vivo model, such as cell–cell interactions, physicochemical gradients, glucose decrease, and oxygen availability [67]. Considering the above, we decided to analyze the changes in the growth of spheroids exposed to AHEE for 48 h and in their reversion to 2D cultures.

**Figure 6:**
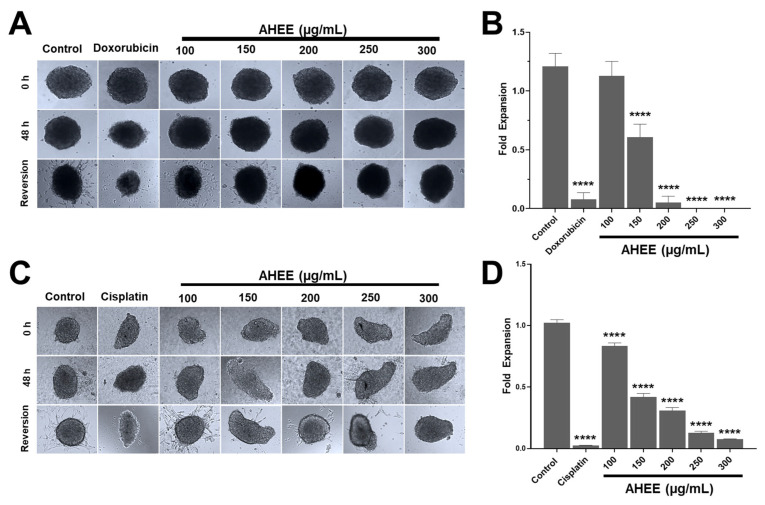
AHEE affects the cancer hallmarks of 3D cultures of TNBC and tumorigenic HPV-16+-related cell lines in a concentration-dependent manner. (**A**) Three-dimensional cultures of MDA-MB-231 cells showed no evident changes in growth after 48 h of treatment with the extract compared to doxorubicin. When the spheroids were seeded on an adherent surface, their conversion to 2D monolayers decreased in a concentration-dependent manner. Magnification 10×. (**B**) The area of reversion to 2D was used as a measure of the extract’s effect on viability and the ability of cells to adhere and migrate. In MDA-MB-231 cells, the reversion declined from 200 µg/mL and was inhibited at 250 µg/mL. (**C**,**D**) The 3D cultures of TC-1 cells also did not show changes in growth, but their conversion to 2D monolayers decreased from 150 µg/mL and dropped drastically at 250 µg/mL. Magnification 10×. **** *p* < 0.0001, compared to the control with medium alone.

No significant changes in spheroid growth were observed between control and extract-treated spheroids in either cell line; however, a decrease in spheroid size was evident in spheroids treated with cisplatin and doxorubicin (Figure 6). As a strategy to establish whether AHEE exposure affected the cells in 3D culture, we analyzed whether cells could adhere, grow, and expand in 2D culture, i.e., revert to an adherent surface, and the growth-expansion area was measured.

The control MDA-MB-231 3D cultures had an expansion fold of 1.21 ± 0.11, while in those exposed to 100 µg/mL of AHEE this was 1.13 ± 0.12, with no statistical difference between them (*p* > 0.9837). Instead, at 150 µg/mL of the extract, the value of the area decreased to about half the size of the control, at 0.61 ± 0.11; at 200 µg/mL it was 0.05 ± 0.05; and at 250 and 300 µg/mL expansion was fully inhibited. These changes were statistically different from untreated spheroids (*p* < 0.0001), whereas compared to doxorubicin, the effect of 200 µg/mL of AHEE showed no statistical difference, with *p*-values of >0.9999, as well as 250 µg/mL (*p* = 0.9648), and 300 µg/mL (*p* = 0.9717) (Figure 6A,B).

The decrease in growth expansion of 3D cultures of TC-1 cells treated with AHEE, in contrast, was statistically different in all the concentrations tested compared to the control (*p* < 0.0001). Specifically, the growth-expansion value of the control was 1.024 ± 0.02; instead, in 3D cultures exposed to 100 µg/mL it was 0.83 ± 0.03; at 150 µg/mL it was 0.42 ± 0.03; at 200 µg/mL it was 0.31 ± 0.02; at 250 µg/mL it was 0.13 ± 0.01; and at 300 µg/mL it was 0.08 ± 0.00. Likewise, the inhibitory effect of the extract at 300 µg/mL was similar to that of cisplatin, whose growth-expansion value was 0.02 ± 0.00, showing no statistical difference compared to cisplatin (*p* = 0.5663), that is, with an equivalent effect to that of the chemotherapeutic agent (Figure 6C,D).

Considering these results, we could suggest that AHEE induces changes in the cells that go beyond affecting the surface cells of the spheroid, i.e., it could diffuse into the spheroid core and trigger signals that are transferred and ultimately lead to altered cancer hallmarks or its inhibition and death of the spheroid-forming cells.

### 3.8. The Combination of AHEE and Low Doses of Chemotherapeutics Decreased MDA-MB-231 and TC-1 Cell Viability

Finally, we sought to determine whether combining the extract with a low dose of cisplatin or doxorubicin could have an additive effect that would decrease the viability of MDA-MB-231 and TC-1 cells. To test this possibility, one-fifth of the IC_50_ dose of each chemotherapeutic agent was used and combined with the extract concentrations tested to establish whether they could decrease viability by 50% or more.

The cytotoxicity of the one-fifth IC_50_ dose of doxorubicin in MDA-MB-231 cells was 21.61% ± 4.05, and this value increased to 52.45%± 0.80 when used in conjunction with the 100 µg/mL concentration of the extract, to 54.18% ± 2.83 with 150 µg/mL, to 60.81% ± 1.09 with 200 µg/mL, to 68.38% ± 0.39 with 250 µg/mL, and to 73.24% ± 4.26 with 300 µg/mL.

**Figure 7:**
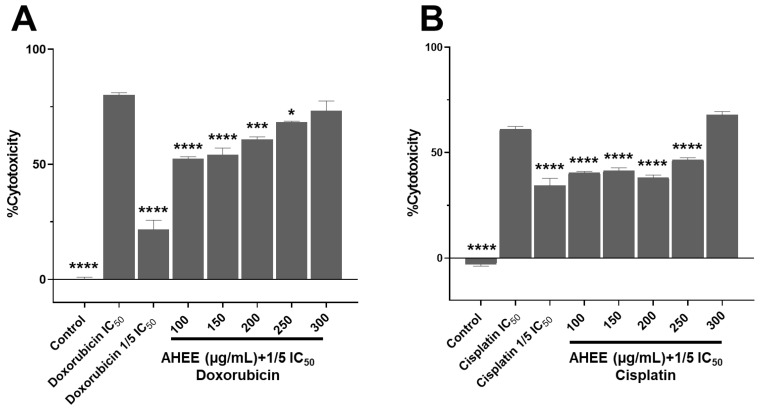
Changes in the cell viability in TNBC and tumorigenic HPV-16+-related cell lines induced by the treatment with the combination of AHEE and low doses of chemotherapeutics. (**A**) MDA-MB-231 cells were incubated for 48 h with combinations of AHEE and 1/5 of the IC_50_ doxorubicin dose. The combination of 100 µg/mL of AHEE and doxorubicin had a cytotoxic effect of 52%; at 250–300 µg/mL, the effect was similar to that of the full IC_50_ dose. (**B**) In TC-1 cells, only the combination of 300 µg/mL of the extract and 1/5 of the IC_50_ cisplatin dose decreased viability to the same level as the full IC_50_ dose. Statistical comparisons were performed against the IC_50_ values of chemotherapeutics. * *p* < 0.05, *** *p* < 0.001, **** *p* < 0.0001, compared to doxorubicin or cisplatin IC_50_ treatments.

The highest concentration was not statistically different from the cytotoxicity value observed under the experimental IC_50_ conditions of doxorubicin, which resulted in 80.08% ± 1.00 cytotoxicity (*p* = 0.4971). In other words, the combination of one-fifth IC_50_ of doxorubicin and 300 µg/mL of the extract generates an effect very similar to the IC_50_ value (Figure 7A). It should be recalled that the extract alone at 100 µg/mL did not affect cell viability, suggesting a possible synergistic effect when combined with the low dose of doxorubicin, reaching a reduction in viability of 52%.

In contrast, in the TC-1 cell line, only the combination of 300 µg/mL with the reduced dose of cisplatin exceeded the effect of the IC_50_ value of the chemotherapeutic alone, with a cytotoxicity of 67.75% ± 1.78 versus 61.15% ± 1.27, with no significant difference between them (*p* = 0.1447). This value was significantly higher than for one-fifth of the IC_50_ dose of cisplatin, which induced a cytotoxicity of 34.30%± 3.49, with a *p*-value of <0.0001. It is noteworthy that in the previous viability assay, the 300 µg/mL extract alone caused a 52.45% reduction in viability, while its combination with one-fifth of the IC_50_ dose of cisplatin increased the effect by 15% (Figure 7B).

These results suggest that the phytochemicals in AHEE (principally flavonoid-type compounds) could act together with doxorubicin and cisplatin without blocking their anticancer activity, an apparent synergistic interaction with doxorubicin, and one additive, which was less pronounced with cisplatin. However, further studies are needed to establish the exact interaction and its safety.

## 4. Discussion

Traditional medicine remains integral in many cultures, particularly in oncology care in western Mexico, where plant-based remedies are frequently used alongside or as alternatives to conventional treatments such as chemotherapy and radiotherapy [68]. One such plant is *A. heterophyllus*, commonly known as jackfruit. In this study, we address two aspects of the biological activity of jackfruit leaves that, to our knowledge, have not been previously analyzed: (1) the phytochemical profile of AHEE, and (2) its anticancer activity in models of triple-negative breast cancer and HPV-16-related cervical cancer.

Initially, we performed a phytochemical study of AHEE. Ethanol was selected due its broad acceptance in herbal medicine and its endorsement in international guidelines for primary screening of plant-based compounds [17]. Preliminary phytochemical screening of AHEE from Colima, Mexico, revealed significant amounts of tannins, steroids, and flavonoids, with smaller amounts of alkaloids, consistent with earlier studies that report flavonoids and phenolic compounds in jackfruit leaves [69,70,71].

AHEE exhibited strong antioxidant capacity, as evidenced by DPPH radical scavenging, total antioxidant capacity, and ferric reducing power assays confirming the previous reports of jackfruit’s high free radical scavenging activity [72,73]. These properties are likely attributed to phenolic metabolites, known to mitigate oxidative stress and regulate cancer-related signaling pathways [74].

TLC and spectroscopic analyses (UV-Vis, IR, and NMR) indicated the presence of flavonoids structurally similar to artocarpin and artocarpetin, which are known for their cytotoxic and anti-inflammatory activities. Other studies on *A. heterophyllus* extracts, using solvents such as water, methanol, ethyl acetate, and dichloromethane extracts, have also reported rich compositions of flavonoids, tannins, saponins, and terpenoids, with various pharmacological properties including cardioprotective, diuretic, hypoglycemic, and even antiviral effects, such as anti-hepatitis C virus activity [75]. Moreover, the safety profile of leaf extracts has been supported by acute and sub-chronic toxicity studies in animal models, which found no significant alterations in hematological, biochemical, or histological parameters [76].

The in vitro evaluation allowed us to assess its effect on cell viability, proliferation, migration, and anchorage-independent growth, incorporating both 2D and 3D culture systems. In the first we evaluated viability. The results showed that AHEE modestly reduced viability in MDA-MB-231 cells (maximum 24%), and more significantly in TC-1 cells (up to 50%.) This differential sensitivity may be due to distinct doubling times and metabolic rates, as MDA-MB-231 cells divide every 25–30 h compared to 18.5 h for TC-1 cells, possibly rendering TNBC cells less susceptible to treatment [77,78]. Similar observations have been reported with licoflavone C, a flavonoid compound isolated from the *Artocarpus* genus, and preliminarily identified in our extract. This compound has shown a pronounced toxicity in metabolically active H4IIE hepatoma and metabolically poorly active C6 glioma cells [79,80,81], while showing cytoprotective effects on human peripheral lymphocytes against the chromosome damage induced by mitomycin C or daunorubicin. In this line, other studies like Liu et al. have analyzed leaf extracts (in this case, combined with root and extracted in methanol) [22], when they isolated seven chromones and five flavonoids, with cytotoxic activity in breast cancer cell line MCF7, hepatocarcinoma cell line SMMC-7721, leukemia cell line HL-60, pancreatic cancer cell line SW480, and lung cancer cell line A-549, and not in the macrophage cells RAW 264.7. The fact that these previously obtained results contrast with the results obtained in our study suggests that these extracts are complex mixtures whose individual components may have specific biological activities. In our extract, either the mixture or the predominant compounds appear to exert a specific anti-proliferative effect.

In addition to these findings, several reports describe flavonoids from *Artocarpus* species with anti-proliferative activity. For example, artocarpin (isoprenyl flavonoid) from the wood of *A. heterophyllus* has been shown to bind directly to Akt 1 and 2 kinases and induces cell cycle arrest in G1 phase in DLD1, HCT116, HCT15, HT29, and SW48 human colon cancer cell lines, and A549, H226, and H1299 non-small cell lung cancer cells [81,82]. Similarly, other *Artocarpus* species like *A. tonkinensis* and *A. lacucha* yielded flavonoids and 2-arylbenzofurans that reduce cell viability in cancer lines MCF-7, SMMC-7721, HL-60, SW480, and melanoma line A-375 [80], and ovarian teratocarcinoma cell line CH1/PA-1 and colon carcinoma cell line SW480 [83]. These studies support the hypothesis that AHEE evaluated under our conditions possibly is rich in this kind of molecule, explaining the modest effects on viability but the strong effects of proliferation.

In migration assays, AHEE’s effect also differed between cell lines, with a greater impact on TC-1 cells. This could be explained considering that MDA-MB-231 cells are highly metastatic, that is, they have increased biological mechanisms that allow them to migrate and invade tissues, such as a high expression of metalloproteinases to degrade the extracellular matrix [77]. Although the extract reduced their migratory capacity, this inhibition was not as pronounced as in the case of TC-1, which, although proven to be metastatic, are not particularly so [78]. The only precedent, to our knowledge, regarding the migration and phytochemicals of the *Artocarpus* genus is a work with the flavonoid norcycloartocarpin isolated from the heartwood of *A. gomezianus*, which significantly inhibited cell motility, at 24 h, of A549, H460, H23, and H292 lung cancer cell lines through the suppression of the FAK/Akt signaling pathway [84]. This suggests that AHEE may contain flavonoids with activity similar to norcycloartocarpin.

Due to the importance of migration in metastasis development, as in the case of MDA-MB-231 cells, it would be interesting to establish the biological targets that could regulate the bioactive molecules present in the extract. Studying the gene expression or the amount of N-cadherin protein, which is upregulated in invasive carcinomas, of the transcription factors Snail, Slug, Twist, and Zeb1/2, and of matrix-degrading enzymes, would help to understand and establish whether there is indeed a specific effect [85]. Up to this point, interestingly, previous in silico studies have shown that artocarpin may interact directly and possibly inhibit the metalloproteases MMP9 and MMP13, suggesting that indeed the compounds present in the leaves may be regulating key factors involved in migration [86,87]. Although this compound comes principally from the wood, it leads us to believe that there may indeed be phytochemicals in the extract that regulate matrix degradation, because artocarpin was preliminary identified in our extract [88].

Anchorage-independent growth, a hallmark of in vivo metastasis, was also inhibited by AHEE (300 μg/mL), particularly in MDA-MB-231 cells, whose low cell–cell adhesion may contribute to their greater sensitivity. In this line, cell aggregation was impaired at low extract concentrations, as was its reversion to 2D cultures.

This difference may be explained by the fact that MDA-MB-231 cells, despite their high metastatic potential, do not form compact tumors like TC-1 cells, which better withstand higher extract concentrations [77,78]. During this biological process, cells undergo a series of changes such as resistance to cell death, reorganization of the cytoskeleton, changes in metabolism, and the production of reactive oxygen species [66,89]. Four factors are key to this process—IKZF1, NFE2, BTG2, and IRF8—and the signaling pathways integrin/FAK/SFKs, Ras/ERK, PI3K/AKT, Rho, and YAP/TAZ [66,89]. Therefore, analyzing their status during treatment with AHEE is the next step toward establishing its mechanism of action on anchorage-independent cell growth inhibition. In this regard, it has been reported that the flavonoids artocarpin and norcycloartocarpin, from the *Artocarpus* genus, inhibited this hallmark of cancer in the colorectal cancer lines HCT115 and HT29, and the lung cancer cell lines A549 and H460, respectively, by directly targeting Akt 1 and 2 kinases [84]. This suggests that there could indeed be flavonoids in AHEE leaves with this activity.

Three-dimensional cultures are a model used to assess drug activity as a preliminary step to in vivo models. Among other processes, they allow the assessment of cell–cell interactions and physicochemical gradients [67]. In this work, the 3D cultures were exposed to AHEE, and their ability to adhere, proliferate, and migrate on an adherent surface were subsequently determined. With this model we confirmed that AHEE exerted inhibitory effects on the cell clusters, suggesting that it could diffuse into the core or trigger signaling in outer cells, which was then transmitted and led to the observed biological responses of anti-adhesion, anti-proliferation, and anti-migration, similar to doxorubicin and cisplatin. This allows us to propose that the extract would have a high probability of being biologically active in an in vivo model. Hence, its evaluation in an animal model is the next step in our line of research. Similarly, we observed that the effect was greater on 3D cultures of MDA-MB-231 cells than on those of TC-1 cells. This differential effect may be due to the greater tumor-forming capacity of TC-1 cells, making the extract’s effect more evident at higher concentrations.

Finally, it was determined whether the extract could be used as an adjuvant to chemotherapy. To this end, its effect on the viability of combined treatments with doxorubicin or cisplatin was analyzed. The results of these experiments were particularly interesting, as they revealed distinct interactions between the chemotherapeutic agents and the extract. In the case of the combination with a low dose of doxorubicin, equivalent to one-fifth of the IC_50_, a synergistic effect appears to be established. AHEE at low doses alone had little effect on MDA-MB-231 cell viability. However, when combined with one-fifth of the IC_50_ dose of doxorubicin, it significantly enhanced cytotoxicity, nearly doubling the expected efficacy. In contrast, with cisplatin, increased cytotoxicity was observed only when combined with the highest concentration of the extract, suggesting an additive rather than synergistic effect. This differential behavior may be related to the phytochemical composition of the extract, as revealed by the preliminary analysis (presence of isoprenyl flavonoids) [90,91]. It is plausible that these compounds modulate cellular pathways differently depending on the chemotherapeutic agent involved, enhancing the efficacy of doxorubicin through mechanisms that might include increased oxidative stress or apoptosis induction, while their interaction with cisplatin is less pronounced. Further mechanistic studies are warranted to clarify these interactions.

In summary, AHEE clearly demonstrated anticancer and antitumor effects. Although these effects varied depending on the cancer cell type, the extract effectively reduced proliferation, migration, and anchorage-dependent growth in both 2D and 3D cultures of highly metastatic and tumorigenic cells. Importantly, it interacted with chemotherapeutic agents without diminishing their activity; rather, it enhanced their cytotoxic effects in certain combinations.

However, our study has limitations. While preliminary analyses indicated the possible presence of flavonoid compounds, we did not perform advanced chromatographic techniques such as HPLC or GC-MS to confirm and quantify them. As for the biological activity of the extract, one limitation is that we did not use non-cancerous cells in our assessments, but we can argue that previous works have established selective effects on cancer cells and not on normal ones, such as CCD-18Co from normal colon epithelium or HPAEpiCs cells from lung epithelium [81,82]. Also, as noted earlier, some reports have established the safety of aqueous and methanolic extracts of the leaves in in vivo models [76,92]. Understanding the precise roles of these compounds will be essential for future development of the extract or its components as adjuvants in cancer therapy. An additional limitation is that we did not analyze human cervical cancer; however, we consider our results to be valid and to set a precedent for more specific analyses. Furthermore, the use of the TC-1 cell line is justified by its usefulness for analyzing the immune response in murine models, an important aspect that will be analyzed subsequently in the lines of work that are opened from these results in our work group [93]. Likewise, it is a limitation that we did not establish a mechanism of action, but our future lines of research include secondary screening of the extract to identify its most active parts and its mechanisms of action. Another restriction is that we did not evaluate the effect of the extract in an animal model; however, the three-dimensional culture is a valid approach, although further experiments are undoubtedly necessary in subsequent preclinical phases to establish whether this can be an applicable treatment for cervical cancer. Future research should address these limitations and build on the current findings to fully explore the therapeutic potential of AHEE.

## 5. Conclusions

The ethanolic extract of *Artocarpus heterophyllus* leaves from Colima, Mexico, contains flavone-type compounds and demonstrated significant anticancer and antitumor activity in both 2D and 3D cultures of the triple-negative breast cancer (TNBC) cell line MDA-MB-231 and the HPV-16+ tumorigenic murine cancer cell line TC-1. The extract notably inhibited cell proliferation, migration, and anchorage-independent growth, suggesting the presence of phytochemicals capable of modulating key hallmarks of cancer.

This study provides a foundation for future research in two main areas: (1) the isolation and detailed characterization of the active compounds responsible for these effects, and (2) elucidation of their molecular mechanisms of action. Additionally, preclinical studies are needed to evaluate the extract’s safety and efficacy in vivo. Given its synergistic interaction with doxorubicin and additive effect with cisplatin, the extract shows promise as a potential adjuvant in cancer therapy.

In summary, our findings highlight the therapeutic potential of *A. heterophyllus* leaves, both as a source of bioactive compounds and as a basis for future exploration of novel anticancer mechanisms and treatment strategies.

## Figures and Tables

**Table 1:** Preliminary phytochemical analysis of the ethanolic extract of *A. heterophyllus* leaves.

Metabolites	AHEE
Tannins (FeCl_3_)	+++
Tannins (gelatin hydrolysis)	+
Flavonoids (Shinoda test)	+++
Flavonoids (Salkowski test)	+++ (Chalcones)
Steroids	+++
Alkaloids (Dragendorff test)	+
Alkaloids (Wagner test)	+
Alkaloids (Mayer test)	−
Saponins (hemolysis in agar)	−
Saponins (foam formation)	−
Coumarins (NaOH test)	+
TFC ^a^	QE = 0.45 ± 0.02 mg/g extract
FRPA ^b^	percentage reduction = 34.09 ± 0.24
TAC ^c^	percentage TAC = 81.25 ± 4.25
TPC ^d^	GAE = 3.34 ± 0.01 µg/mg extract
DPPH ^e^	Scavenging effect = 86.34 ± 4.18%

+++ appreciable amount (positive within 5 min); + = trace amount (positive after 10 min but within 15 min); − = completely absent. ^a^ TFC (total flavonoid content): expressed in quercetin equivalents (QE) µg/mg of extract. ^b^ FRPA (ferric reducing power assay): expressed as % reducing power relative to the ascorbic acid control. ^c^ TAC (total antioxidant capacity): expressed as % antioxidant capacity relative to the ascorbic acid control. ^d^ TPC (total phenolic content): expressed in µg of gallic acid (GAE) per mg of extract. ^e^ DPPH: scavenging effect (%). Concentration at 1.25 mg/mL.

**Table 2:** Anti-browning results mean ± standard deviation and the post hoc Tukey test.

	Overall Color Difference (∆E)^2^ Mean ± SD	Control	AHEE 1.0 mg/mL	AHEE 0.5 mg/mL	Ascorbic Acid
post hoc *p*-values at hour 24					
Control	20.41 ± 3.41		<0.001	<0.001	<0.001
AHEE 1.0 mg/mL	14.98 ± 1.60	<0.001		ns	<0.001
AHEE 0.5 mg/mL	13.64 ± 1.60	<0.001	ns		<0.001
Ascorbic acid	8.28 ± 1.48	<0.001	<0.001	<0.001	
P (ANOVA)	<0.001				
post hoc *p*-values at hour 36					
Control	27.19 ± 2.32		ns	<0.001	<0.001
AHEE 1.0 mg/mL	20.53 ± 0.61	<0.001		ns	<0.001
AHEE 0.5 mg/mL	17.60 ± 3.75	ns	ns		<0.001
Ascorbic acid	10.28 ± 0.67	<0.001	<0.001	<0.001	
P (ANOVA)	<0.001				

Mean ± standard deviation (SD) of (∆E)^2^ values for the effect of AHEE on the browning of freshly cut apple slices over 36 h at 20 °C. Ascorbic acid (5 mg/mL) was used as the positive control for antioxidant activity. All samples were treated in an aqueous solution. Each storage time point includes photographs of apple slices treated with AHEE at 0.5 and 1.0 mg/mL. Statistical significance was determined using Tukey’s post hoc test at a significance level of 0.05. ns = not significant (*p* > 0.05).

## Data Availability

The original contributions presented in the study are included in the article/Supplementary Material; further inquiries can be directed to the corresponding author.

## Supplementary Materials

The following supporting information can be downloaded at: https://www.mdpi.com/article/10.3390/life15071090/s1, Figure S1. Thin-layer chromatography (TLC) analysis of the ethanol extract of AHEE and reference compounds. Figure S2. UV spectra recorded in MeOH at 0.1 mg/mL. Figure S3. Infrared spectra (wavenumber in cm^−1^). Figure S4. ^1^H-NMR spectra (400 MHz) of AHEE, recorded in DMSO-d6. Figure S5. ^13^C NMR spectra (100 MHz) of leaves of AHEE, recorded in DMSO-d6. Figure S6. DEPT135 spectra (100 MHz) of leaves of AHEE, recorded in DMSO-d6. Figure S7. DEPT90 spectra (100 MHz) of leaves of AHEE, recorded in DMSO-d6. Table S1. Comparisons of chemical shifts of AHEE vs. some of the principal metabolites isolated from the leaves of *Artocarpus heterophyllus*.
