# Supplementary material for: Exploring the Anticancer Activity of Artocarpus heterophyllus Leaves: Selective Effects on Triple-Negative Breast Cancer and HPV16-Positive Tumorigenic Cells
Source: Life (Basel). 2025 Jul 11;15(7):1090. doi: 10.3390/life15071090 (PMC12298788; doi:10.3390/life15071090)
Supplement: Supplementary file 1 [file life-15-01090-s001.zip › life-3683295-supplementary.pdf]

Supplementary information

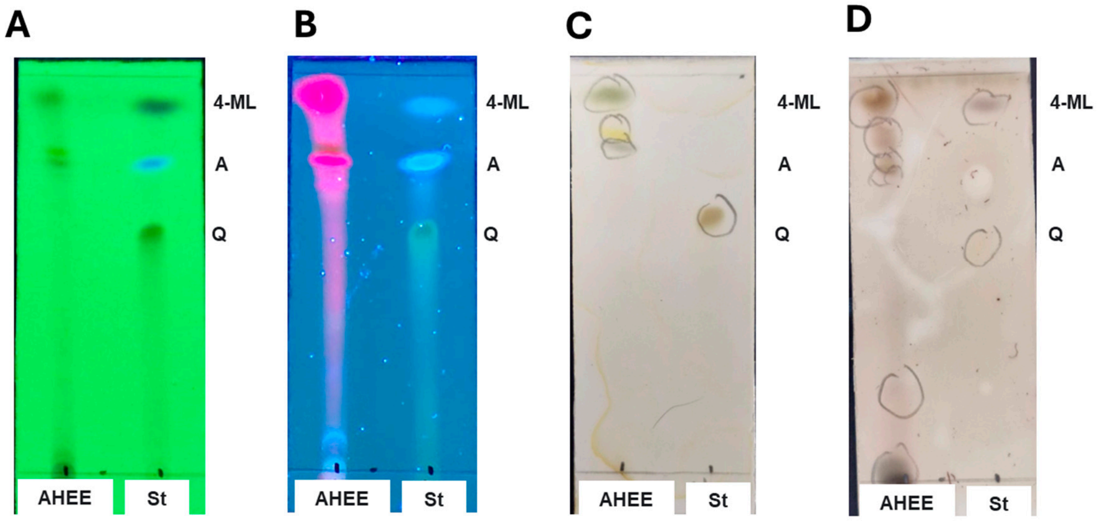

Figure S1. Thin-layer chromatography (TLC) analysis of the ethanol extract of AHEE and reference compounds. The figure displays five TLC plates: (A) chromatography under short-waved UV light (254 nm), (B) chromatography under long-waved UV light (365 nm), (C) visualization with 10% ferric chloride (FeCl), and (D) visualization with ceric sulphat. The samples analyzed include AHEE: ethanol extract of A. heterophyllus (1.0 mg/mL). St (Standarts Q: quercetin (1 mg/mL), A: anthrone (1 mg/mL), and 4-ML: 4-methylumbelliferone (1 mg/mL). The solvent system used was 8:2 chloroform/methanol).

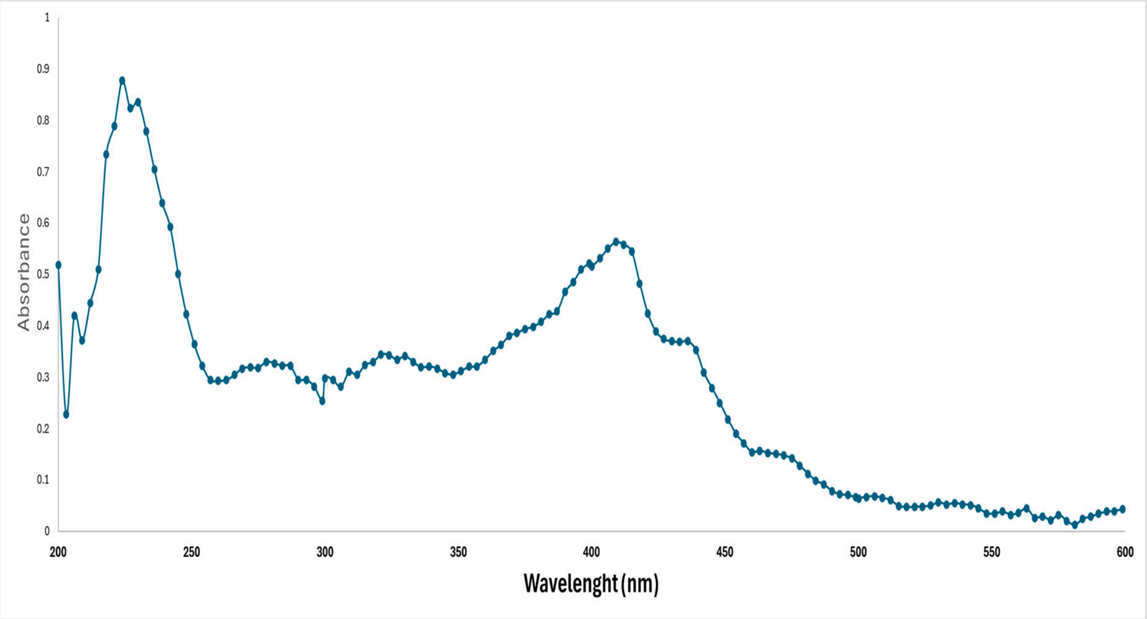

Figure S2. UV spectra recorded in MeOH at 0.1mg/mL.

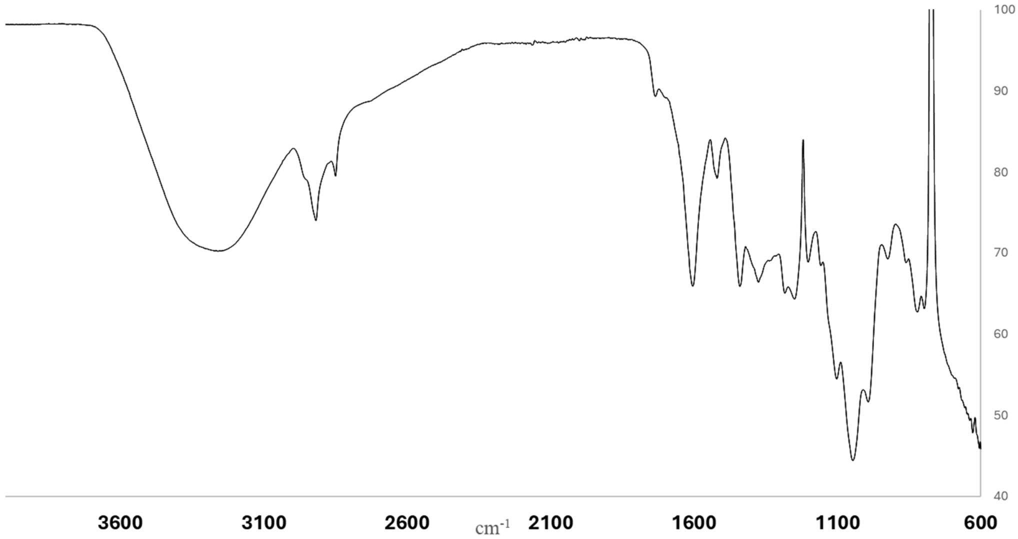

Figure S3. Infrared spectra (wavenumber in cm^{-1})

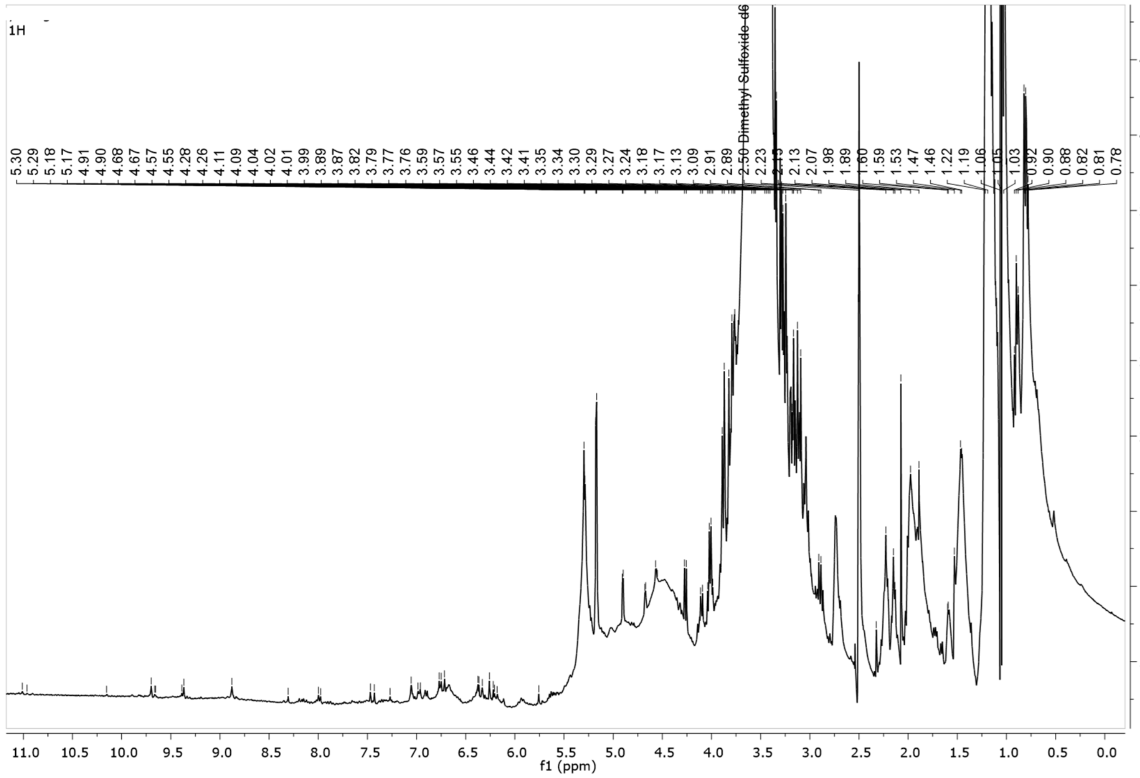

Figure S4. [1] H-NMR spectra (400 MHz) of AHEE, recorded in DMSO-*d6*

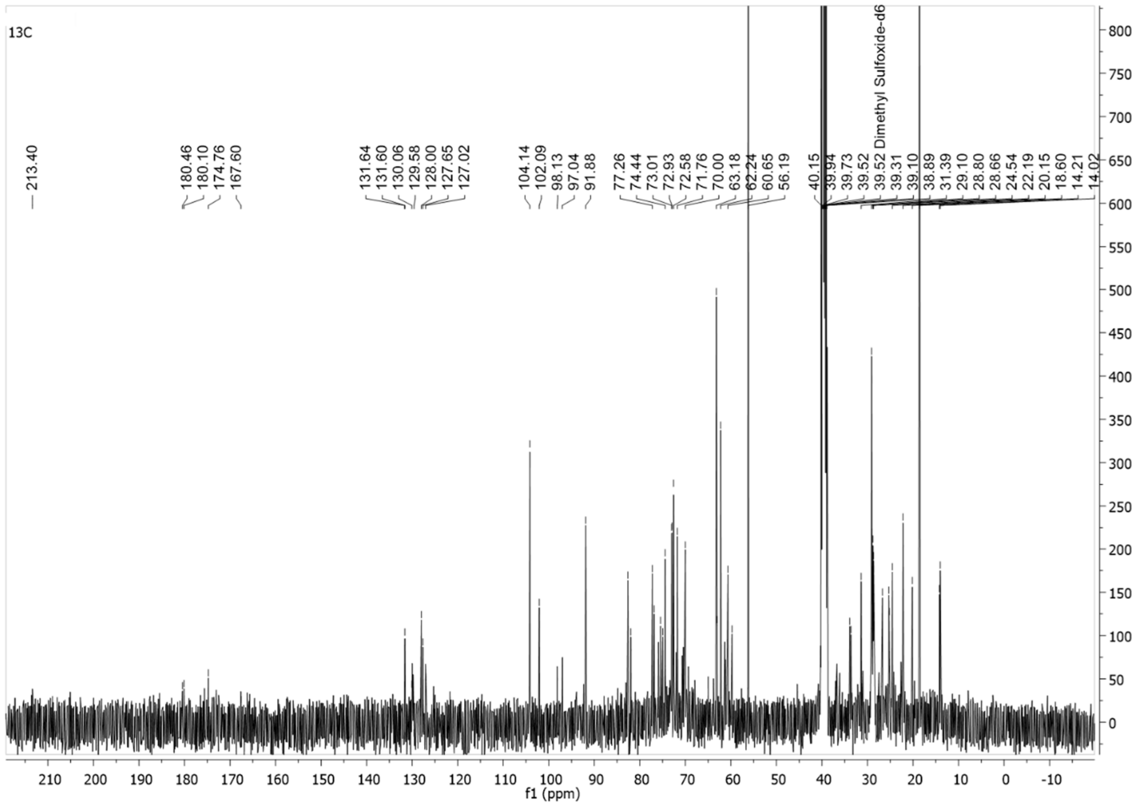

Figure S5. ^{13}C NMR spectra (100 MHz) of leaves of AHEE, recorded DMSO-*d6*

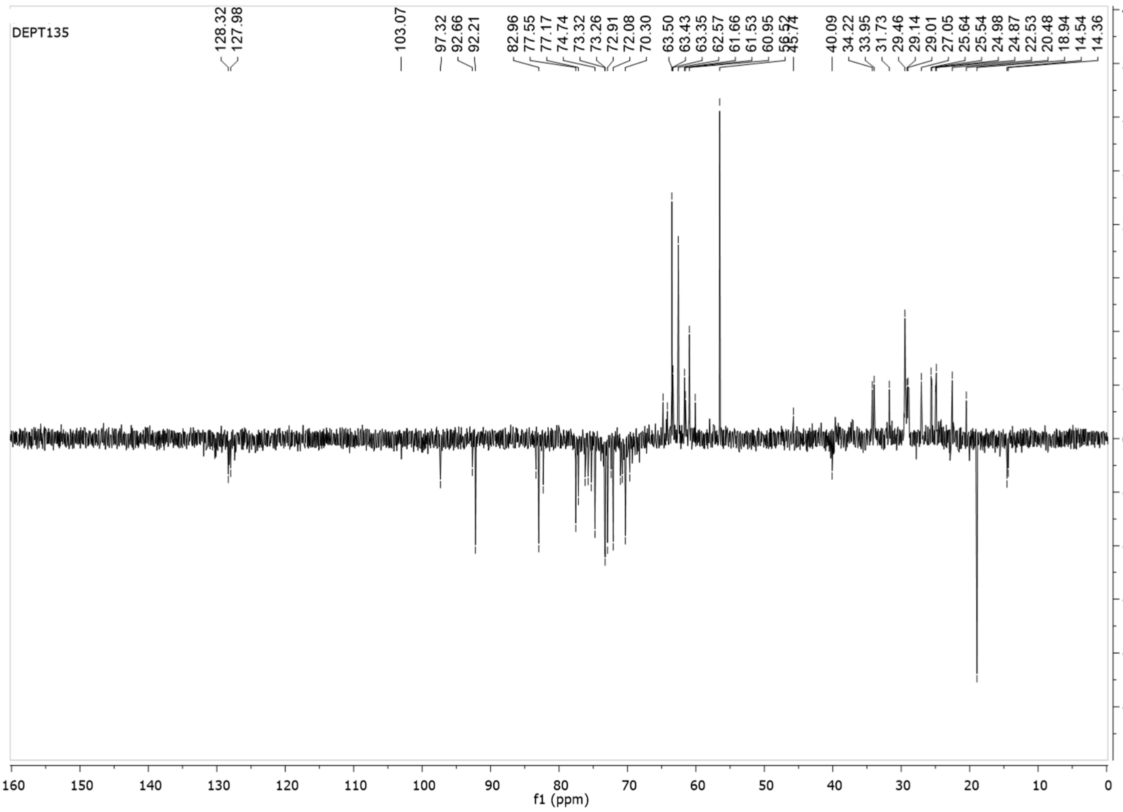

Figure S6. DEPT135 spectra (100 MHz) of leaves of AHEE, recorded DMSO-*d6*

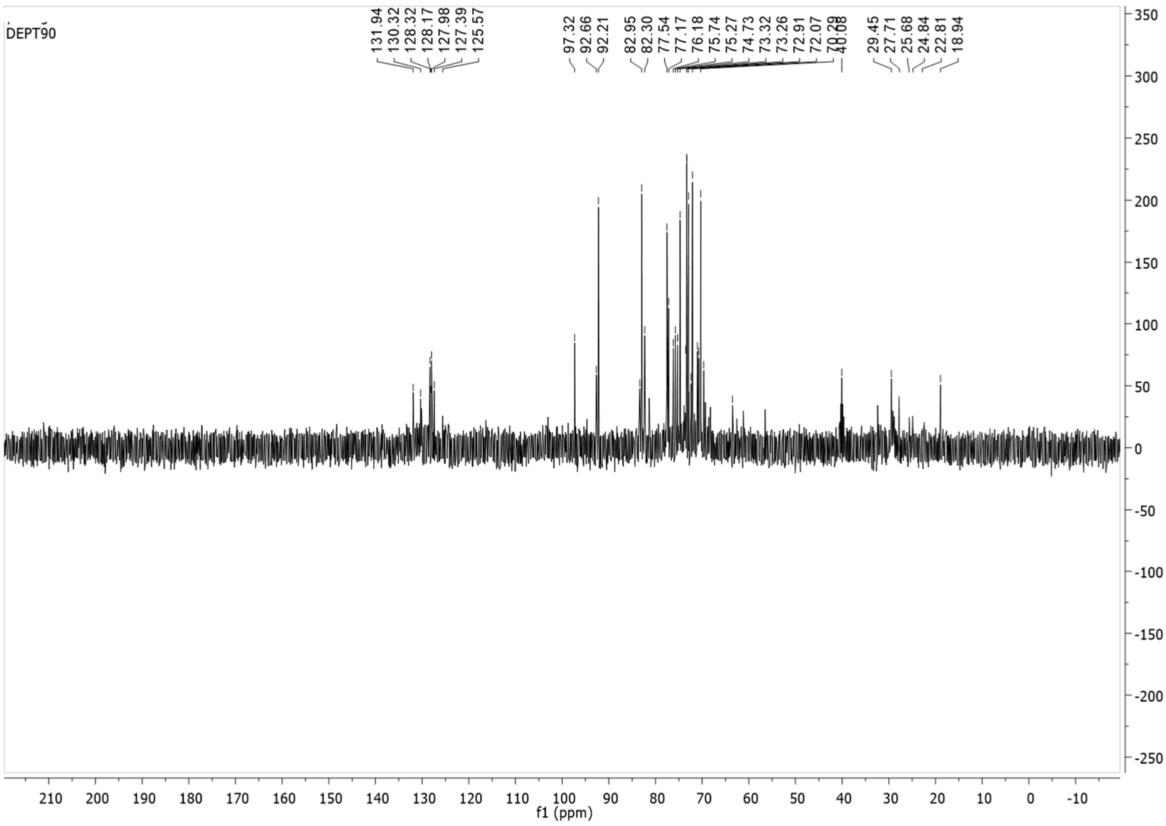

Figure S7. DEPT90 spectra (100 MHz) of leaves of AHEE, recorded DMSO-*d6*

Table S1. Comparisons of chemical shifts of the AHEE vs some of the principal metabolites isolated from the leaves of *Artocarpus heterophyllus*

AHEE*				Asterric Acid*		Licoflavone C**		Artocarpin**		Artocarppanona**	Artocarpetin**
				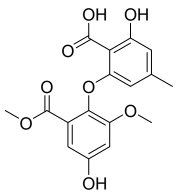 $C_{17}H_{16}O_8$		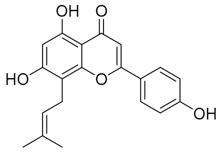 $C_{20}H_{18}O_5$		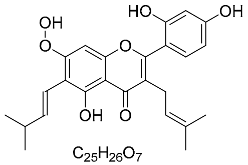 $C_{25}H_{26}O_7$		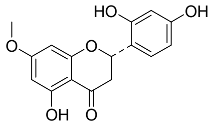 $C_{16}H_{14}O_6$	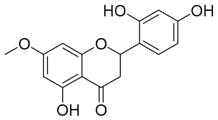 $C_{16}H_{14}O_6$
**^{1}H**	**^{13}C**	**DEPT135**	**DEPT90**	**^{1}H**	**^{13}C**	**^{1}H**	**^{13}C**	**^{1}H**	**^{13}C**	**^{13}C**	**^{13}C**
11.01 *s*	213.40	128.32 CH	131.94		107.3	7.85 *d*	177.30	13.98 *s*	180.3	129.4	163.1
10.96 *s*	180.46	127.98 CH	130.32		156.5	7.0 *d*	163.80	8.84 *s*	159.9	108.3	109.1
10.15 *s*	180.10	103.07 CH	128.32	6.31 *br.s*	109.6	6.5 *s*	163.10	7.24 *d*	122.0	165.4	109.1
9.70 *s*	174.76	97.32 CH	128.17		141.3	6.25 *s*	159.10	6.74 *dd*	105.6	103.9	184.2
9.66 *d*	167.60	92.66 CH	127.98	6.53 *br.s*	104.4	5.25 *t*	153.90	6.62 *d*	142.3	156.7	160.2
9.39-9.37 *d*	131.64	92.26 CH	127.39		157.1	4.90 *s*	132.20	6.58	109.8	117.1	99.8
8.88 *s*	131.60	82.96 CH	125.57		167.1	3.75 *s*	128.40	6.57 *s*	163.4	104.1	156.6
8.31 *s*	130.06	77.55 CH	97.32	3.76 *s*	51.9	3.60 *d*	126.70	6.54 *dd*	90.5	165.1	94.8
8.00-7.98 *d*	129.58	77.17 CH	92.66	2.06 *s*	21.4	3.25 *s*	126.20	5.13 *t*	157.5	95.7	163.3
7.47 *s*	128.00	74.74 CH	92.21		125.7	1.85 *d*	122.70	3.98 *s*	116.2	169.1	105.2
7.43 *s*	127.65	73.32 CH	82.95	6.74 (*d*, 2.9)	107.5	1.75 *s*	122.0	3.14 *d*	142.21	94.8	110.7
7.27 *s*	127.02	73.26 CH	82.30		133.8	1.25 *s*	120.40	2.45	90.6	160.0	159.4

7.06 *s*	104.14	72.91 CH	77.54	6.76 (*d*, 2.9)	104.8	0.9 *t*	117.50	1.59 *s*	132.3	198.5	104.2
6.99 *s*	102.09	72.08 CH	77.17		153.5		115.80	1.45 *s*	24.6	43.0	163.9
9.96 *s*	98.13	70.30 CH	76.18		155.2		104.70	1.10	122.5	75.9	106.4
6.77 *s*	97.04	40.09 CH/ CH_3	77.54		165.4		104.10		121.9		131.0
6.75 *s*	91.88	18.94 CH_3	77.17	3.61 *s*	52.1		100.60		25.8		
6.72 *s*	77.22	14.54 CH_3	76.18	6.39 *s*	56.1		73.30		17.6		
6.38 *s*	74.44	14.33 CH_3	75.74				50.80		117.0		
6.37 *s*	73.01	63.50 CH_2	75.27				28.60		122.6		
6.33 *s*	72.93	63.43 CH_2	74.75				25.90		34.0		
6.26 *s*	72.58	63.35 CH_2	73.32				25.30		23.1		
6.22 *s*	71.76	62.57 CH_2	73.26				18.20		23.1		
6.21 *s*	70.00	61.66 CH_2	72.91						56.6		
6.18 *s*	63.18	61.53 CH_2	72.07								
5.76 *s*	62.24	60.95 CH_2	72.07								
5.30 *s*	60.55	56.52 CH_2	70.29								
5.29 *s*	56.19	34.22 CH_2	40.08								
5.18 *s*	40.65	33.95 CH_2	29.45								

5.17 *s*	39.94	31.73 CH_2	27.71								
4.91-4.01*m*	39.73	29.46 CH_2	25.61								
3.99-3.13*m*	39.52	29.14 CH_2	24.84								
2.89 *s*	39.31	29.01 CH_2	22.81								
2.90-2.07 *br.s*	39.10	27.05 CH_2	19.94								
1.98-0.88 *br.s*	38.89	25.64 CH_2									
0.81 *s*	31.39	25.54 CH_2									
0.78 *s*	29.10	24.98 CH_2									
	28.80	24.87 CH_2									
	28.66	22.53 CH_2									
	24.54	20.41 CH_2									
	24.19										
	20.15										
	18.60										
	14.21										
	14.02										

δ from TMS (ppm). ^{1}H NMR [400 MHz, J (Hz)] and ^{13}C NMR (100 MHz) data for AHEE. *br* (broad signal), *s* (singlet), *m*(multiplet). ** Acetone-d6 as a solvent. [a] DEPT135 experiment. [b] DEPT90 experiment.Chemical shifts obtained from x (40,41), Y (40,41) and Z (42). *DMSO-d6 as a solvent.
